# The clinical impact of copy number variants in inherited bone marrow failure syndromes

**DOI:** 10.1038/s41525-017-0019-2

**Published:** 2017-05-10

**Authors:** Nicolas Waespe, Santhosh Dhanraj, Manju Wahala, Elena Tsangaris, Tom Enbar, Bozana Zlateska, Hongbing Li, Robert J. Klaassen, Conrad V. Fernandez, Geoff D. E. Cuvelier, John K. Wu, Yves D. Pastore, Mariana Silva, Jeffrey H. Lipton, Joseé Brossard, Bruno Michon, Sharon Abish, MacGregor Steele, Roona Sinha, Mark J. Belletrutti, Vicky R. Breakey, Lawrence Jardine, Lisa Goodyear, Liat Kofler, Michaela Cada, Lillian Sung, Mary Shago, Stephen W. Scherer, Yigal Dror

**Affiliations:** 1 Genetics and Genome Biology Program, The Hospital for Sick Children, Toronto, ON Canada; 2 Marrow Failure and Myelodysplasia Program, Division of Hematology/Oncology, Department of Pediatrics, The Hospital for Sick Children, Toronto, ON Canada; 3 Institute of Medical Sciences, University of Toronto, Toronto, ON Canada; 4 Department of Pediatrics, Children’s Hospital of Eastern Ontario, Ottawa, ON Canada; 5 Pediatric Hematology/Oncology, IWK Health Centre, Halifax, NS Canada; 6 Pediatric Hematology/Oncology, University of Manitoba, CancerCare Manitoba, Winnipeg, MB Canada; 7 Division of Hematology/Oncology, UBC & B.C. Children’s Hospital, Vancouver, BC Canada; 8 CHU Sainte-Justine, Montreal, QC Canada; 9 Kingston General Hospital, Kingston, ON Canada; 10 Allogeneic Blood and Marrow Transplant Program, Princess Margaret Cancer Centre, University of Toronto, Toronto, ON Canada; 11 Centre Hospitalier Universitaire, Sherbrooke, QC Canada; 12 Centre Hospitalier Universitaire, Québec, QC Canada; 13 Pediatric Hematology/Oncology, Montreal Children’s Hospital, Montreal, QC Canada; 14 Alberta Children’s Hospital, Calgary, AB Canada; 15 Royal University Hospital, Saskatoon, SK Canada; 16 Department of Pediatrics, University of Alberta, Edmonton, AB Canada; 17 Department of Pediatrics, McMaster University, Hamilton, ON Canada; 18 Children’s Hospital, London Health Sciences Centre, London, ON Canada; 19 Pediatric Hematology/Oncology, Janeway Child Health Centre, St. John’s, NF Canada; 20 Population Health Sciences, Research Institute, Division of Hematology/Oncology, Department of Pediatrics, The Hospital for Sick Children, Toronto, ON Canada; 21 Cytogenetics Laboratory, Department of Paediatric Laboratory Medicine, The Hospital for Sick Children, Toronto, ON Canada

## Abstract

Inherited bone marrow failure syndromes comprise a genetically heterogeneous group of diseases with hematopoietic failure and a wide array of physical malformations. Copy number variants were reported in some inherited bone marrow failure syndromes. It is unclear what impact copy number variants play in patients evaluated for a suspected diagnosis of inherited bone marrow failure syndromes. Clinical and genetic data of 323 patients from the Canadian Inherited Marrow Failure Registry from 2001 to 2014, who had a documented genetic work-up, were analyzed. Cases with pathogenic copy number variants (at least 1 kilobasepairs) were compared to cases with other mutations. Genotype-phenotype correlations were performed to assess the impact of copy number variants. Pathogenic nucleotide-level mutations were found in 157 of 303 tested patients (51.8%). Genome-wide copy number variant analysis by single-nucleotide polymorphism arrays or comparative genomic hybridization arrays revealed pathogenic copy number variants in 11 of 67 patients tested (16.4%). In four of these patients, identification of copy number variant was crucial for establishing the correct diagnosis as their clinical presentation was ambiguous. Eight additional patients were identified to harbor pathogenic copy number variants by other methods. Of the 19 patients with pathogenic copy number variants, four had compound-heterozygosity of a copy number variant with a nucleotide-level mutation. Pathogenic copy number variants were associated with more extensive non-hematological organ system involvement (*p* = 0.0006), developmental delay (*p* = 0.006) and short stature (*p* = 0.04) compared to nucleotide-level mutations. In conclusion, a significant proportion of patients with inherited bone marrow failure syndromes harbor pathogenic copy number variants which were associated with a more extensive non-hematological phenotype in this cohort. Patients with a phenotype suggestive of inherited bone marrow failure syndromes but without identification of pathogenic nucleotide-level mutations should undergo specific testing for copy number variants.

## Blood disorders: impact of genomic structural variation

Copy number variation in patients with inherited bone marrow failure syndromes (IBMFSs) is associated with more severe clinical symptoms. In addition to persistently low levels of red blood cells, white blood cells and/ or platelets, patients with IBMFSs also present varying degrees of physical malformations. Most cases are associated with single base-pair mutations in the DNA sequence, but Canadian researchers led by Yigal Dror at The Hospital for Sick Children in Toronto, have found that whole sections of the genome are deleted or repeated in an important proportion of patients. Those carrying copy number variants (CNV) presented more commonly with developmental delay, short stature and defects in more organ systems, than patients with point mutations. CNV analysis of patients with suspected IBMFSs could aid early disease evaluation and management.

## Introduction

Inherited bone marrow failure syndromes (IBMFSs) are complex genetic disorders characterized by hematopoietic failure, which results in single or multi-lineage cytopenias, and an increased prevalence of physical malformations.^1–3^ A wide variety of specific syndromes have been described so far with more than 80 different genes associated to IBMFSs. These include genes involved in ribosome biogenesis that are associated with Diamond–Blackfan anemia^4^ and Shwachman-Diamond syndrome,^5^ genes involved in telomere maintenance, associated with dyskeratosis congenita,^6^ and genes involved in DNA repair associated with Fanconi anemia.^7^ Pathogenic mutations in these pathways perturb cell homeostasis and lead ultimately to failure of the bone marrow to produce a sufficient amount of blood cells. Based on the inheritance patterns of IBMFSs in multiplex families and the segregation of mutated alleles in known IBMFS genes of phenotypically affected family members, the disorders are considered monogenic in the vast majority of patients.

A large proportion of IBMFSs are important cancer predisposition syndromes, with a particularly increased risk of myelodysplastic syndrome (MDS) and acute myeloid leukemia (AML).^1, 2^ In particular, Fanconi anemia,^8^ Shwachman–Diamond syndrome,^9^ severe congenital neutropenia,^10^ and familial thrombocytopenia with predisposition to MDS/AML^11^ were associated with the development of malignancies. There is an overlap of classical IBMFSs and secondary MDS due to MDS-predisposing germline mutations (e.g., *RUNX1, ETV6*, and *GATA2*-related disorders), as patients with these mutations can present with MDS without any additional features of IBMFSs.^12^ The risk of development of cancers differs greatly between the various IBMFSs, and identification of the underlying etiology of marrow failure is imperative to assess the need and type of cancer screening.^2^

Identifying the causal genotype in cases with IBMFSs is challenging.^13^ In part, the challenge derives from a large number of patients who cannot be classified into specific syndromic diagnoses, in spite of having features highly suggestive of an inherited disorder.^14^ To improve the efficiency of genetic testing, utilization of comprehensive next generation sequencing panels was shown to be successful. In a previous report from our group using a panel of 72 genes, the genotype was elucidated in 59% of the patients with clinically classified disorders, and 18% of the patients with marrow failure who were not clinically classified into one particular IBMFS.^15^

Copy number variants (CNVs, i.e. insertions and deletions of at least 1 kilobasepairs) have been found as the underlying genetic mutation in a variety of disorders. Specifically, neurocognitive illnesses, such as autism spectrum disorders and schizophrenia,^16^ epilepsy,^17^ cerebral palsy,^18^ and congenital heart disease^19, 20^ were found to be associated with this type of genetic alterations. Short stature has also been associated with rare CNVs.^21^ Cases of IBMFSs with pathogenic CNVs have been reported previously, most notably in Diamond–Blackfan anemia^22–24^ but also in Fanconi anemia.^25, 26^ In particular, an association of these types of mutations with neurodevelopmental delay^22^ and short stature^23^ has been suggested in small series of patients with Diamond–Blackfan anemia.

Identification of CNVs is not efficiently done through Sanger sequencing and targeted next-generation gene panel sequencing.^27^ Therefore, CNVs are often missed when a genome-wide analysis, such as a single nucleotide polymorphism (SNP) array or comparative genomic hybridization (CGH) array, is not performed.^28–30^ The cost of these tests is relatively modest around USD 300–400 per assay. In contrast, commercially available targeted next-generation sequencing gene panels, used to detect smaller aberrations of the genetic code, harbor costs of around USD 1500–4000 per assay.^15^

The proportion of cases harboring pathogenic CNVs among patients who have, or are suspected to have IBMFSs, and the importance of finding such genetic lesions in this population is unknown. The aims of this study were to determine the utility of genome-wide microarray analysis to detect pathogenic CNVs, to characterize the genetic lesions and phenotypes of patients with CNVs, and to identify clinical features that might be associated with CNVs. Since CNVs directly disrupt all or a large sequence of the respective gene, and sometimes adjacent genomic regions, it was hypothesized that patients with pathogenic CNVs would likely have a more extensive clinical phenotype compared to nucleotide-level mutations (i.e., single nucleotide variants and small insertions/deletions).

## Results

### Characteristics of the patients

Of the 451 patients enrolled in the Canadian Inherited Marrow Failure Registry (CIMFR) from January 2001 to December 2014, genetic test results were available for 323 (71.6%) patients. Genetic testing was done at the discretion of the treating physicians and/or as part of systematic genotyping of enrolled patient into our research laboratory (YD). One hundred eighty of the 323 patients (55.7%) were found to have causal mutations, which included nucleotide-level mutations, CNVs, or both (Supplementary Table 1). Four patients had insufficient data to determine the type of mutation, and they were excluded from further analysis. Patients were diagnosed with a wide variety of IBMFSs according to established criteria (Supplementary Table 2).

### Nucleotide-level variant analysis

Among the group of 323 patients who had genetic testing, 303 patients (93.8%) underwent investigation for nucleotide-level mutations by either Sanger sequencing, disease-specific next-generation sequencing (NGS) panels, or by a comprehensive research panel of 72, and later 77 IBMFS genes (ibmfNGS Panel).^15^ These genetic evaluations were undertaken either at our research laboratory (YD), or accredited clinical laboratories. Causal nucleotide-level mutations were found in 157 patients (51.8%, Supplementary Table 3). These included 37 patients with small indels involving 1–20 bp. The majority of unclassified patients had undergone extensive genetic testing, which was negative.^13–15^

### Genome-wide CNV analysis

Sixty-seven of 323 patients had genome-wide CNV analysis by SNP/CGH arrays (20.7%). Of those, 39 were assessed in a research laboratory (YD) and the rest in a clinical laboratory. The identified median number of CNVs in the patients assessed in the research laboratory was 141 (interquartile range 133–158) per patient. Further filtering identified a median of 65.5 (59–72) CNVs being >1 kb with five or more probes and 5 (3–7) were found to be rare. Finally, zero to four CNVs per patient were found to affect coding regions in all but one patient. The latter was found to have a significantly higher number of rare CNVs, which might be due to ethnical differences.

Of the 67 patients tested with genome-wide CNV analysis, 11 were found to harbor pathogenic CNVs, resulting in a detection rate of 16.4%. Seven patients were identified with a monoallelic CNV (four patients with Diamond-Blackfan anemia and one with Potocki–Lupski syndrome, supernumerary ring chromosome 1 syndrome, and *RUNX1*-associated familial thrombocytopenia/AML, each), one patient (with Fanconi anemia) harbored two different compound-heterozygous CNVs, and three patients were compound-heterozygous for a CNV and a nucleotide-level mutation (one patient with dyskeratosis congenita and two patients with Thrombocytopenia absent radius syndrome, respectively). In four of 11 cases who were genotyped by SNP/CGH array, genotyping was critical for establishing the diagnosis, as their clinical phenotype was not explicit. The diagnoses in these cases included *PARN*-associated dyskeratosis congenita (published previously),^31^ *RPL11*- and *RPL35a*-associated Diamond-Blackfan anemia, and Potocki–Lupski syndrome (Table 1).^32^

**Table 1:** Characteristics of the identified copy number alterations

Patient number	Syndrome	Gene/genomic region (inheritance pattern)	HumanGenome19 coordinates or cytogenetic location	Approximate size of the CNV	Detection method
1#	Diamond–Blackfan anemia	*RPL35a* (AD)	del(chr3:186,550,246–197,837,050)x1	11.3 Mb	Array 1 and FISH
2#	Diamond–Blackfan anemia	*RPS17* (AD)	del(chr15:83,196,738–84,812,671)x1	1.6 Mb	Array 2
3#	Diamond–Blackfan anemia	*RPL5* (AD)	del(chr1:92,932,837–94,018,138)x1	1.1 Mb	Array 3
4#	Diamond–Blackfan anemia	*RPL11* (AD)	del(chr1:23,449,116–24,147,166)x1	698 kb	Array 4
5	TAR syndrome	Chr1q21.1	del(chr1:145,390,101–145,792,052)x1	402 kb	Array 5
		*RBM8A* (AR)	c.−21G>A (chr1:145,507,646G>A)	SNV	
6	TAR syndrome	Chr1q21.1	del(chr1:145,372,550–145,792,064)x1	420 kb	Array 5
		*RBM8A* (AR)	c.−21G>A (chr1:145,507,646G>A)	SNV	
7	TAR syndrome	Chr1q21.1	del(chr1:144,100,000–144,290,000)x1*	190 kb	Quantitative PCR
		*RBM8A* (AR)	NA	SNV	
8	Trisomy 8 syndrome	Chr8 (aneuploidy)	dup(chr8)	145.1 Mb	Cytogenetics and FISH
9	Trisomy 8 syndrome	Chr8 (aneuploidy)	dup(chr8)	145.1 Mb	Cytogenetics and FISH
10	Trisomy 8 syndrome	Chr8 (aneuploidy)	dup(chr8)	145.1 Mb	Cytogenetics and FISH
11#	Fanconi anemia	*FANCA* (AR)	FANCA deletion exon 31*	5 kb	MLPA
			FANCA deletion exons 4–29*	49 kb	MLPA
12#	Fanconi anemia	*FANCA* (AR)	del(chr16:89,799,574–89,847,471)x1	47.9 kb	Array 2
			del(chr16:89,824,684–89,869,755)x1	45 kb	Array 2
13#	Dyskeratosis congenita	*PARN* (AR)	del(chr16:14,658,272–14,679,880)x1	21.6 kb	Array 5
			c.1045C>T (chr16:14,676,047G>A)	SNV	
14#	Familial Thrombocytopenia/AML	*RUNX1* (AD)	del(chr21:34,965,815–36,781,907)x1	1.8 Mb‡	Array 6 and FISH
15	Pearson syndrome	mitDNA (mitochondrial)	del(mitDNA)*	6 kb	Southern blot
16	SRC1 syndrome	Chr1p13.1	+r(chr1:116,673,235–152,748,194)	36.1 Mb	Array 7 and cytogenetics
17	Wolf–Hirschhorn syndrome	Chr4p	der(4)t(4;8)(p16.3;p23.1)*	2.5 Mb(del), 9 Mb(dup)	Cytogenetics and FISH
18	Jacobson syndrome	11q23	del(11)(q23.3)x1*	17 Mb	Cytogenetics
19	Potocki–Lupski syndrome	17p11.2	dup(chr17:16,778,108–18,252,450)x1	1.5 Mb	Array 8 and FISH

#, patients included in the subset analysis; *, for methods without identification of the exact breakpoint, the genomic locations were estimated as well as the total size of the respective CNV; ‡, complex intrachromosomal rearrangement of chromosome 21 including a deletion involving the RUNX1 gene

*AD* autosomal dominant, *AML* acute myeloid leukemia, *AR* autosomal recessive, *Array 1* Agilent 105 K Human Genome Oligonucleotide array, *Array 2* Agilent 180 K Human Genome Oligonucleotide array, *Array 3* GeneDx 180 K microarray v4, *Array 4* Agilent Oligo Array – EmArray cyto 6000 custom design, *Array 5* Affymetrix SNP Array 6.0, *Array 6* CytoSure Syndrome Plus V2, *Array 7* Roche NimbleGen 135 K oligonucleotide array, *Array 8* Signature Genomic SignatureChipWG Whole genome BAC array, *FISH* fluorescence in-situ hybridization, *MLPA* Multiplex Ligation-dependent Probe Amplification, *NA* not available, *SNV* single nucleotide variant, *SRC1* supernumerary ring chromosome 1, *TAR* thrombocytopenia absent radius

In addition to the 11 patients identified with genome-wide CNV analysis, patients were also found to have pathogenic CNVs by metaphase cytogenetics, multiplex ligation-dependent probe amplification assay, fluorescence in-situ hybridization, DNA-quantitative real-time PCR analysis, and Southern blotting (Supplementary Table 3). Altogether, 19 patients were found to harbor pathogenic CNVs in our study. The CNVs ranged from 5 kb to 145.1 Mb in size (Table 1). The most common disease associated with pathogenic CNVs in this population was Diamond–Blackfan anemia, found in four of 35 genotyped patients (11.4%); followed by thrombocytopenia absent radius syndrome (*n* = 3, 100%), trisomy 8 syndrome (*n* = 3, 100%), and Fanconi anemia found in two of 28 patients (7.1%). Four patients had compound heterozygosity for a CNV and nucleotide-level mutations (Supplementary Table 1).

### Differences in clinical phenotypes between patients with CNVs and nucleotide-level mutations

**Fig. 1:**
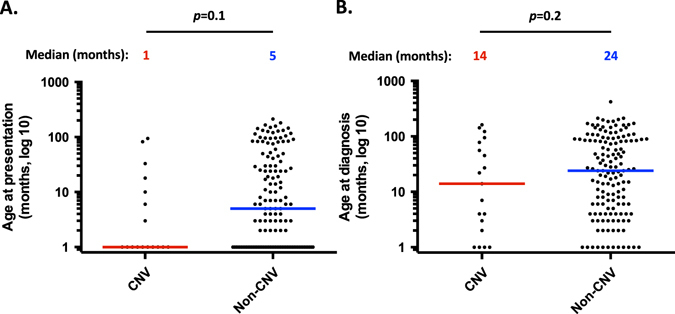
Analysis of clinical parameters comparing cases with CNVs vs. other mutations. **a** Age at presentation, color bars representing corresponding median; **b** Age at diagnosis, color bars representing corresponding median

The clinical characteristics of patients who harbor pathogenic CNVs or nucleotide-level mutations are shown in Table 2. Demographic data were similar among the groups. The age at presentation and age at diagnosis were assessed where available, which was the case in 17 and 19 patients harboring CNVs, respectively, and 143 and 146 patients without CNVs, respectively. There was no statistical difference between cases with CNVs and cases with nucleotide-level mutations (Fig. 1).

**Table 2:** Phenotypic characteristics of patients included in this study, sorted by cases with copy number variants (CNVs) and other mutations

	Patients with CNVs	Patients with other mutations	*p-*value
	(*n* = 19)	(*n* = 157)	
Sex (females, %)	7 (36.8%)	76 (48.4%)	*p* = 0.5
Median age at presentation (IQR)	1 month (1–14)	5 months (1–41)	*p* = 0.1
Median age at diagnosis (IQR)	14 months (2–78)	24 months (5–88.25)	*p* = 0.2
Median age at last follow up (IQR)	12.6 years (6.5–16.7)	12.2 years (7–18)	*p* = 0.6
Severe anemia (*n*, %)	9 (50%)	64 (46.4%)	*p* = 0.8
NA (*n*)	1	19	
Severe neutropenia (*n*, %)	5 (35.7%)	56 (41.8%)	*p* = 0.8
NA (*n*)	5	23	
Severe thrombocytopenia (*n*, %)	5 (31.3%)	18 (13.3%)	*p* = 0.07
NA (*n*)	3	22	
Severe aplastic anemia (*n*, %)	4 (21.1%)	33 (21%)	*p* = 1.0
NA (*n*)	0	0	
MDS/ AML (*n*, %)	5 (26.3%)	26 (16.7%)	*p* = 0.3
NA (*n*)	0	1	
Median number of non-hematological systems involved (IQR)	6 (3.75–8)	3 (1–5)	*p* = 0.0006
Developmental delay (*n*, %)	11 (61.1%)	40 (27.4%)	*p* = 0.006
NA (*n*)	1	11	
Short stature (*n*, %)	12 (66.7%)	59 (40.1%)	*p* = 0.04
NA (*n*)	1	10	

*CNV* copy number variant, *IQR* inter-quartile range, *NA* data not available

**Fig. 2:**
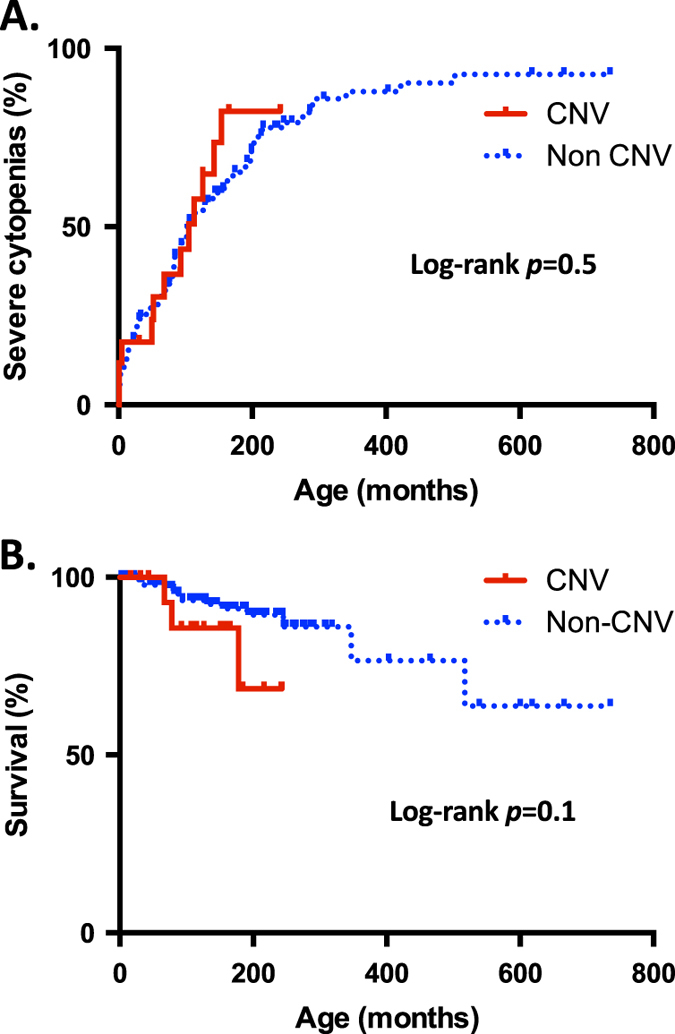
Analysis of onset of hematological complications and survival comparing cases with CNVs vs. other mutations. **a** Age at onset of severe cytopenias; **b** Overall survival

To assess the potential effect of pathogenic CNV mutations on hematological phenotype across the different types of IBMFSs, the age at onset of severe cytopenias was assessed where this information was available. No differences were found between patients with CNVs (*n* = 17) and those without CNVs (*n* = 146) (Fig. 2a). Similarly, the overall survival was not different between the two groups (Fig. 2b).

**Fig. 3:**
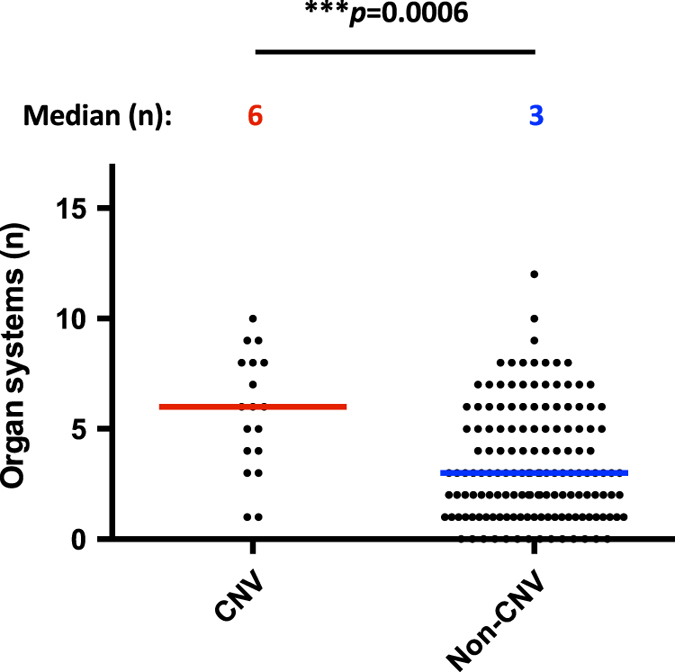
Analysis of non-hematological organ system involvement comparing cases with CNVs vs. other mutations. Color bars representing corresponding median

**Fig. 4:**
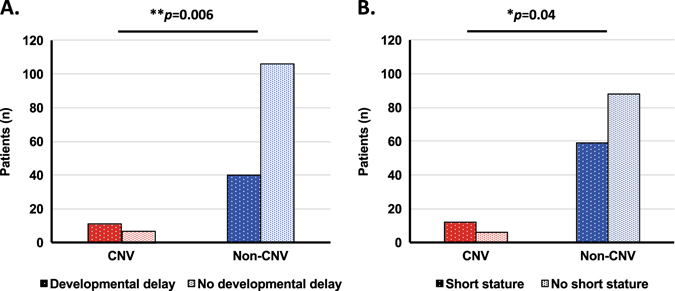
Analysis of specific organ system involvement comparing cases with CNVs vs. other mutations. **a** Developmental delay; **b** Short stature

The correlation of CNV mutations with the extent of involvement of non-hematological organ systems was analyzed. Agreement in assignment of non-hematological organ system involvement between two evaluators was excellent (kappa = 0.87; 95%-CI 0.85–0.89). Patients with pathogenic CNVs were found to have symptoms in significantly more non-hematological organ systems compared to patients with nucleotide-level mutations (*p* = 0.0006; Fig. 3). Patients with CNVs were also found to have a significantly higher proportion of developmental delay (*p* = 0.006) and short stature (*p* = 0.04) compared to patients with nucleotide-level mutations (Fig. 4).

Since the above analyses included several syndromes with small numbers of patients, a subgroup analysis of only IBMFSs with at least five cases that included patients with CNVs, as well as patients with nucleotide-level mutations, was performed. Patients with Diamond–Blackfan anemia, Fanconi anemia, dyskeratosis congenita, and familial thrombocytopenia/AML and MDS predisposition syndromes due to *RUNX1, ANKRD26* gene mutations, and *GATA2*-associated MDS/AML met these criteria. Patients harboring whole or large segmental chromosome alterations that are typically identified through metaphase cytogenetics and patients with thrombocytopenia-absent radius syndrome who are typically identified by combined sequencing and targeted copy number analysis of the *RBM8A* gene were thus excluded. Patients included in this sub-analysis were all identified by genome-wide CNV analysis. This subgroup analysis revealed no differences between patients with CNVs (*n* = 8) and nucleotide-level mutations (*n* = 81) with regard to age at presentation, age at diagnosis, onset of severe cytopenias, and overall survival (Supplementary Figs. 1 and 2). However, similar to the whole group analysis, a significantly higher number of organ system involvement was found among cases with CNVs compared to other types of mutations (*p* = 0.007). Importantly, a significantly higher incidence of developmental delay (*p* = 0.04), and short stature (*p* = 0.02) was also found in the subgroup analysis.

Finally, to further adjust for potential bias caused by unequal distribution of syndromes with high or low number of organ system involvement between the two genetic groups, the extent of non-hematological organ system involvement was dichotomized. A trend was seen towards more cases showing “extensive non-hematological organ system involvement” for the specific syndromes with CNVs (75%) compared to those without (39%) but the difference in incidence did not reach statistical significance (*p* = 0.07).

## Discussion

Patients who present with bone marrow failure often cannot be directly assigned to a specific syndromic and genetic diagnosis. Our group and others showed that utilization of a comprehensive nucleotide-level testing approach, that targets a large number of known IBMFS genes in one next-generation sequencing panel, have resulted in a high identification rate of causal mutations with sometimes unexpected genetic findings and amendment of clinical diagnoses.^15, 33^ The present study shows that genome-wide CNV analysis can reveal causal mutations in a substantial number of patients with IBMFSs. Importantly, there was a difference in non-hematological phenotypes in patients affected with pathogenic CNVs compared to other types of mutations.

It is well known that patients with nucleotide-level mutations in IBMFS genes can express hematological and non-hematological symptoms as a consequence of a single gene mutation. It is unknown what role CNVs play in IBMFSs as a group. The CIMFR database offers the opportunity to assess a large cohort of patients with IBMFSs in Canada. The registry data reflects the unselected spectrum of diseases that get attention for workup of a suspected IBMFS. The vast majority of patients was tested for nucleotide-level mutations which revealed the causal genetic germline mutation in about half of the patients. The current analysis included 67 patients tested with a genome-wide approach to identify CNVs. Of those, 16.4% harbored a pathogenic CNV, which helped establish the genetic diagnosis in this patient cohort.

Depending on the source of DNA, somatic mutations can contaminate germline DNA sequences. The present study reduced the risk of falsely identifying mutations as being germline by the choice of sampled cells (see Methods section). Nevertheless, in a small number of patients, peripheral blood myeloid cells were not removed from the analysis and a possibility of somatic CNVs cannot be completely excluded.

Previous studies assessed the extent of non-hematological symptom manifestations by simply counting the number of congenital defects.^34^ Since patients with IBMFSs may have multiple defects in only one organ system (e.g., café-au-lait spots, hyperpigmentation, and leukoplakia on the tongue and mucosal surfaces), this approach does not provide information about the potential biological links between a genetic lesion and the extent of involvement of different organ systems. To overcome this limitation, the symptoms were grouped by affected organ system and the number of organ systems summed up. It was previously unknown whether the presence of pathogenic CNVs increases the risk of having more extensive organ system involvement. In this study, patients with CNVs were found to be affected in significantly more non-hematological organ systems than those with only nucleotide-level mutations. This was true for the whole cohort including patients with structural chromosomal changes. Interestingly, this association remained true for a subgroup of IBMFSs where at least five patients were present with at least one patient harboring a CNV excluding structural chromosomal changes, rare IBMFSs, and IBMFSs where no CNVs were present.

Short stature and developmental delay are common features among patients with IBMFSs. These complications are primarily skeletal and neurological abnormalities, respectively. However, both might be affected by defects in various other organ systems (e.g., gastrointestinal, endocrine, cardiac etc.). We speculated that larger mutations (i.e., CNVs) might affect several areas of the genome, influencing several organ systems and therefore have an effect on development and stature as markers for multisystem involvement.

Furthermore, there is previously published data showing that CNVs might be associated with a higher incidence of developmental delay and short stature in patients with Diamond–Blackfan anemia but no such association was seen in other IBMFSs.^22, 23^ Developmental delay and short stature were therefore analyzed separately. Both were found to be more prevalent in patients with CNVs in the whole cohort but also in the subgroup analysis of patients with at least 5 patients and CNVs present. Due to the sample size, it was difficult to assess the impact of CNVs on specific IBMFSs. Nevertheless, it is noteworthy that among patients with Diamond-Blackfan anemia, three of four patients with CNVs had growth retardation and three of four had developmental delay (vs. 35.5% and 51.6% of patients without CNVs, respectively).

Assessment of the age at presentation, age at diagnosis and age at onset of severe cytopenias was performed. Most patients included in this study had this information available. In contrast to the non hematological phenotype in the same patient group, there was no difference in the age at presentation, age at diagnosis and the age at onset of severe cytopenias between patients harboring CNVs and those who do not have this type of mutation. This might be due to the hematological phenotype being influenced exclusively by the gene associated with the IBMFS and not adjacent genomic areas. Since IBMFSs are rare, the number of patients assessed might also have influenced the lack of difference. Future studies with larger patient groups might elucidate if there is a genuine difference in the mechanisms of the hematological and non hematological phenotype in patients affected with CNVs.

No attempt has previously been undertaken to assess the impact of CNVs on the diagnostic investigative work-up of patients who have signs of IBMFSs. Furthermore, for the entire group of IBMFSs, there has not been a comparative assessment of CNVs yet. To our knowledge, this is the largest study in which a comprehensive analysis of various IBMFSs with known causal genotypes has been conducted.

Our study has several limitations. First, although 19 patients with CNVs is a relatively large number when rare disorders such as IBMFSs are analyzed, larger studies are necessary to validate these results. Specifically, the number of patients per IBMFS was too small for intrasyndromic variation analysis. Another limitation is that patients harboring compound-heterozygous and homozygous mutations have been analyzed together in autosomal recessive diseases. In such cases, the genotype severity was graded according to the presumably most damaging lesion, i.e., CNVs. It would be of interest to analyze these groups separately to assess whether there are differences in cases with heterozygous, homozygous and compound-heterozygous CNV mutations. In addition, some mutations (e.g., nonsense or indel with frameshift) can have a major impact on the pathophysiology of the disease by either completely abrogating the translated protein or changing its function. The impact of a variant on the function of the translated protein was not assessed in this analysis. Similarly, some missense mutations have major deleterious effects on protein function and these mutations can cause a severe clinical phenotype. One such example is the Hoyeraal-Hreidarsson syndrome associated with *TINF2* mutations.^35^ Further, IBMFS genes may vary in the degree of essentiality and affect the phenotype in different ways. Last, it is noteworthy that our approach to quantify organ system involvement could not assess the impact of encountered defects on morbidity and quality of life. Future methods that focus on these questions should be developed.

The mechanisms for the encountered differences in CNVs compared to other mutations could not be elucidated with this study. CNVs could affect the phenotype of IBMFS patients by either severely damaging the IBMFS protein itself or by affecting additional genes surrounding the IBMFS gene locus. Future research needs to evaluate what mechanism is underlying this difference in phenotypes.

In summary, most patients with IBMFSs have nucleotide-level mutations. However, an important proportion of patients harbor pathogenic CNVs that are not efficiently detected by current nucleotide-level testing methods. Therefore, genome-wide CNV analysis should be considered in IBMFS cases where nucleotide-level sequencing does not reveal the causal mutation or in cases with autosomal recessive disorders where only one pathogenic allele was found by nucleotide-level analysis. According to our analysis this is true not only for Diamond–Blackfan anemia and Fanconi anemia patients as suggested previously,^22–26^ but in general for patients with suspected IBMFS. The costs associated with this additional analysis are relatively modest. In urgent cases, simultaneous investigation to reveal both, nucleotide-level mutations and CNVs, should be considered. In this IBMFS cohort, pathogenic CNVs were associated with more extensive non-hematological organ system involvement, and a higher prevalence of developmental delay and short stature. Patients with these clinical features should be assessed with CNV analysis early in the evaluation of their disease.

## Methods

### The Canadian inherited marrow failure registry

The Canadian Inherited Marrow Failure Registry (CIMFR) is a multicenter prospective study, which was approved by the Research Ethics Boards of all participating institutions. Individuals who fulfilled diagnostic criteria for an IBMFS^13^ were recruited by pediatric hematologists and oncologists at participating centers who care for >95% of the eligible pediatric IBMFS population in Canada. Patients were enrolled in the study registry after informed written consent was obtained from the patient or their guardian.

Eligibility criteria included evidence of chronic bone marrow failure in addition to either a family history of an IBMFS, physical malformations, presentation earlier than 1 year of age, or positive genetic testing for an associated IBMFS gene. When possible, each case was assigned a specific syndromic diagnosis by the participating center. Diagnoses were reviewed centrally, and if necessary were adjusted based on published diagnostic criteria of specific IBMFSs^1, 3, 36, 37^ after verification with the respective center. Cases that fulfilled the eligibility criteria, but did not meet the clinical, laboratory and genetic diagnostic criteria for any known IBMFS subtype were defined as unclassified IBMFSs.^14^ Patients were excluded if they were diagnosed with (i) acquired aplastic anemia (i.e., without the above criteria for inherited bone marrow failure), (ii) de novo leukemia or leukemia with an unclassified syndrome without preceding bone marrow failure, or (iii) de novo myelodysplastic syndrome (MDS).

The current study included patients who had been enrolled in the CIMFR from its inception in January 2001 until December 2014. Data available for these patients until June 2015 were analyzed. Patient information at study entry and at yearly follow-up was collected and included demographics, clinical symptoms, family history, physical malformations, laboratory tests, diagnoses, genetic tests, imaging studies, treatment, and outcomes.

### Next generation sequencing panel assay and Sanger sequencing

Genomic DNA from either bone marrow fibroblasts, expanded peripheral blood T-cells, or whole blood cells was extracted. Comprehensive testing for 72 and 77 IBMFS genes respectively was done by an NGS IBMFS gene panel using the HaloPlex Capture Kit (Agilent Technologies, Santa Clara, CA) for DNA library preparation, and Illumina HiSeq 2500 platform for sequencing, as described previously.^15^ Sanger sequencing was done as previously described.^13^ Briefly, targeted genes were analyzed by bidirectional sequencing of individual exons and flanking intronic regions after PCR amplification.

### Genome-wide copy number variant analysis

For genome-wide CNV analysis, Affymetrix SNP6.0 array, Affymetrix Inc., Santa Clara, CA, was performed in our research laboratory (YD). To minimize the possibility of detecting somatic CNVs, genomic DNA was extracted from either bone marrow fibroblasts, expanded peripheral blood T-cells, or whole blood cells. The assay features 1.8 million genetic markers, including more than 906,600 SNPs and more than 946,000 probes for detection of CNVs. It was performed according to the manufacturer’s instructions as previously reported.^9^ A subset of patients were analyzed with other CNV arrays in one of two clinical laboratories: either the clinical Cytogenetics laboratory at the Hospital for Sick Children (Toronto, Ontario, Canada), or at Prevention Genetics (Marshfield, Wisconsin, USA; Table 1 and Supplementary Table 4). In these cases, DNA from either phorbol myristate acetate-stimulated lymphocytes or whole blood was extracted and standard protocols were used for the assays and variant calling.

Three different types of CNV calling algorithms were used, namely Birdsuite,^38^ iPattern,^39^ and Affymetrix Genotyping Console software. CNVs that were more than 1 kb in size with five or more consecutive array probes were selected. CNVs seen in 2 or 3 algorithms were considered as stringent CNVs and were included in further analysis. CNVs were further filtered based on their occurrence in healthy control datasets that were provided from The Center of Applied Genomics at the Hospital for Sick Children. CNVs that were seen frequently in healthy controls were removed and only rare CNVs were further prioritized based on (1) CNV type (either deletion or gain), (2) region affected (coding or non-coding regions of the gene), (3) CNVs affecting individual genes vs. multiple genes and (4) known function of the gene in hematopoiesis.

### Metaphase cytogenetics and fluorescence in situ hybridization

Metaphase cytogenetic analysis and fluorescence in situ hybridization (FISH) were performed as previously described.^40^ In brief, for metaphase cytogenetics, bone marrow or peripheral blood cells were cultured and stopped in metaphase, stained and assessed by light-microscopy and computer-based imaging techniques. FISH was performed with the locus-specific probe according to clinical suspicion of a specific gene being affected.

### Grouping of cases according to genetic mutations

A genetic variant was called “causal” if the aberration fulfilled criteria of being “most likely pathogenic” by in silico analysis^15^ or previously described as “pathogenic” in public databases with a corresponding clinical phenotype. Patients were grouped according to the causal mutation having one of the following types of mutations: (1) CNVs spanning at least 1 kb,^41^ (2) all other types of genetic mutations (i.e., single nucleotide variants, and indels). All cases were grouped by the causal genetic mutation type in dominantly inherited diseases. In diseases with recessive inheritance, cases were assigned as follows: at least one pathogenic CNV qualified for analysis in group 1; all other mutations in group 2.

### Assessment of hematological and non-hematological phenotype

To analyze the impact of different genetic aberrations on the disease phenotype across all IBMFSs, age at presentation, age at diagnosis, age at onset of severe cytopenias, and overall survival were analyzed. Age at presentation was defined as occurrence of any symptoms attributed to an IBMFS of the hematopoietic or any other organ system. Age at diagnosis was set as the age at which the specific IBMFS was diagnosed either clinically or by genetic testing. Age at onset of severe cytopenias was the age at which either of the following peripheral blood count values were noted: (i) hemoglobin of <70 g/L and/or need for transfusions or other treatments to increase the hemoglobin (e.g., steroids) for at least 3 months; (ii) platelet count of <20 × 10^9^/L and/or need for platelet transfusions for at least 3 months; (iii) absolute neutrophil count <0.5 × 10^9^/L and/or need for granulocyte colony-stimulating factor treatment for at least 3 months. Overall survival (OS) was defined as the time from birth to last follow up (censored) or death from any cause (event).

As various organ systems are affected in IBMFSs, the extent of non-hematopoietic organ system involvement was evaluated. The absolute number of organ systems affected was assessed by grouping clinical symptoms into 17 different organ systems, and evaluating the number of organ systems affected by at least one symptom (Supplementary Table 5). Acquired symptoms and complications (e.g., acute infections) were excluded. The involvement of each organ system was assigned by two independent investigators (NW, MW). Agreement in assignment was evaluated using the kappa statistic. Discordant findings were reviewed and differences were resolved in a discussion among three investigators (NW, MW, YD).

Cases were considered having “extensive non-hematological organ system involvement” if non-hematological organ system involvement exceeded the median found for the assigned IBMFS in the present study. “Limited non-hematological organ system involvement” was defined as having an equal or lower number of organ systems affected compared to the median for the specific syndrome.

Short stature was defined as body height below the fifth percentile for age. Developmental delay was defined as a delay in the attainment of developmental milestones for young children and/ or inability to attend regular school without special assistance for school-aged children.

### Statistical analysis

Results were presented using descriptive statistics, including calculation of median and inter-quartile ranges (IQR) for continuous variables, and percentages for categorical variables. Fisher’s exact test was used to compare dichotomous variables. Mann–Whitney U test was used to assess for differences in continuous variables. Survival analysis and evaluation of onset of severe cytopenias were performed with the Kaplan Meier estimate and Mantel–Cox log-rank test. Two-tailed *p* < 0.05 was considered statistically significant. Statistical analysis was conducted through Graphpad Prism 6.0 h (La Jolla, California, USA).

### Data availability

As many of the IBMFSs included in this study contain only small numbers of individual patients, the risk of identifying single patients is high despite avoidance of explicit identifiers. Therefore, the clinical data are not publicly available due to them containing information that could compromise research participant privacy/consent.

## Electronic supplementary material

Supplementary Data

Supplementary Figures
