# Supplementary material for: The clinical impact of copy number variants in inherited bone marrow failure syndromes
Source: NPJ Genom Med. 2017 May 10;2:18. doi: 10.1038/s41525-017-0019-2 (PMC5498150; doi:10.1038/s41525-017-0019-2)
Supplement: Supplementary file 1 — Supplementary Data [file 41525_2017_19_MOESM1_ESM.pdf]

Supplementary Data

The clinical impact of copy number variants in inherited bone marrow failure syndromes

Nicolas Waespe, Santhosh Dhanraj, Manju Wahala, Elena Tsangaris, Tom Enbar, Bozana Zlateska, Hongbing Li, Robert J. Klaassen, Conrad V. Fernandez, Geoff D. E. Cuvelier, John K. Wu, Yves D. Pastore, Mariana Silva, Jeffrey H. Lipton, Joseé Brossard, Bruno Michon, Sharon Abish, MacGregor Steele, Roona Sinha, Mark J. Belletrutti, Vicky R. Breakey, Lawrence Jardine, Lisa Goodyear, Liat Kofler, Michaela Cada, Lillian Sung, Mary Shago, Stephen W. Scherer, and Yigal Dror.

Supplementary Table 1: Characteristics of the identified causal genetic changes.

Type of mutation		Patients (n)
CNV		
	- heterozygous	13
	- homozygous or compound-heterozygous	2
CNV & missense/ promotor		3
CNV & not further specified second mutation		1
Nonsense		
	- heterozygous	18
	- homozygous	1
Nonsense & indel (frameshift)		2
Nonsense & splicing/ missense		30
Insertion/ deletion (indel) (frameshift)		
	- heterozygous	23
	- homozygous	3
Indel (frameshift) & splicing/ missense		8
Splicing		
	- heterozygous	11
	- homozygous	4
Splicing & missense		4
Indel (inframe)		1
Missense		
	- heterozygous	43
	- homozygous	9
Unclear mutation type		4
**Total**		**180**

Supplementary Table 2: Diagnoses of included patients who had genetic testing for copy number variants, nucleotide level mutations, or both.

	**Inherited Bone Marrow Failure Syndrome**	**Number of patients with identified causal genotype**
1.	Diamond-Blackfan anemia	35
2.	Shwachman-Diamond syndrome	35
3.	Fanconi anemia	28
4.	Dyskeratosis congenita	22
5.	Kostmann syndrome/ severe congenital neutropenia	10
6.	Cyclic neutropenia	8
7.	Familial platelet disorder with predisposition to AML and AML-/ MDS-predisposition syndromes (*RUNX1, ANKRD26, ETV6, DDX41, SRP72, CEBPA,* and *GATA2* gene-related)	5
8.	Glycogen storage disease Ib	5
9.	Barth syndrome	4
10.	Thrombocytopenia absent radius (TAR)	3
11.	Familial macrothrombocytopenia, *MYH9*-related disorders	3
12.	Trisomy 8 syndrome	3
13.	Familial non-syndromic thrombocytopenia	2
14.	Congenital amegakaryocytic thrombocytopenia	2
15.	Congenial dyserythropoietic anemia	2
16.	Inherited sideroblastic anemia	2
17.	WHIM syndrome (warts, hypogammaglobulinemia, immunodeficiency, myelokathexis)	2
18.	*ERCC6L2*-associated aplastic anemia	1
19.	Pearson syndrome	1
20.	4p-deletion syndrome	1
21.	Supernumerary ring chromosome 1 (SRC1) Syndrome	1
22.	Jacobsen syndrome	1
23.	*WAS*-associated inherited neutropenia	1
24.	Cohen syndrome	1
25.	Potocki-Lupski syndrome	1
26.	Grey platelet syndrome	1
**Total**		**180**

Supplementary Table 3: List of the various methods used to genotype the patients in the study and number of the patient who were tested positive.

**Testing type details**	**Number of patients tested**	**Number of positive results#**	**Comments**
**1. Nucleotide level sequencing analysis (Sanger, disease specific panels and comprehensive ibmfsNGS panel, Dror lab)**	303	157	24 of the 157 patients were also tested by method 2<br>7 of the 157 patients were also tested by method 3<br>114 of the 157 patients were also tested by method 4
**2. Genome-wide CNV analysis (SNP array and/ or CGH array)***	67	11	7 of the 11 patients were also tested by method 1<br>3 of the 11 patients were also tested by method 3<br>8 of the 11 patients were also tested by method 4
**3. Targeted CNV analysis**<br><br>**a. FISH**<br>**b. MLPA**<br>**c. Southern Blot**<br>**d. qPCR**	28<br><br>19<br>3<br>5<br>2	10<br><br>7<br>1<br>1<br>1	2 of the 10 patients were also tested by method 1<br>3 of the 10 patients were also tested by method 2<br>4 of the 10 patients were also tested by method 4
**4. Metaphase cytogenetics**	247	8	4 of the 8 patients were also tested by method 1<br>4 of the 8 patients were also tested by method 2<br>6 of the 8 patients were also tested by method 3
**Total number of tests from all the above approaches that have been performed**	645	186	
**Total number of patients**	323	180*	

*, overall, there were 157 patients whose identified causal genetic lesions included nucleotide-level mutations only, 15 who had CNVs only, and 4 patients who had compound heterozygosity for a CNV and nucleotide level mutations. 4 patients did not have detailed information on the specific mutation type; #, some mutations were identified with more than one test: 3 patients had positive targeted CNV analysis and genome-wide CNV analysis, 7 patients had positive cytogenetics and either targeted or genome-wide CNV analysis; CGH, comparative genomic hybridisation; CNV, copy number variant; FISH, fluorescence in situ hybridisation; ibmfsNGS, inherited bone marrow failure syndromes next-generation sequencing; MLPA, multiplex ligation-dependent probe amplification; qPCR, quantitative polymerase chain reaction; SNP, single nucleotide polymorphism.

Supplementary Table 4: List of the genome-wide copy number variant assays used to genotype the patients in the study.

Assay	Results (n positive/ n tested)
Affymetrix SNP6.0 array	**3/ 44** (7 patients were tested with this assay and another genome-wide approach, see below)
Agilent 180K Oligonucleotide array	**2/ 8**
Agilent 105K Oligonucleotide array	**1/ 6**
Agilent Oligo Array - EmArray cyto 6000 custom design	**1/ 4**
Roche Nimblegen 135K oligonucleotide array	**1/ 4**
Syndrome Plus V2 105k	**1/ 1**
GenomeDx 180K microarray V4	**1/ 1**
Signature Genomic SignatureChipWG Whole genome BAC array	**1/ 1**
Roche Nimblegen CGX-12 array	**0/ 1**
Blue Gnome CytoChip	**0/ 1**
Not further specified whole-genome array	**0/ 3**
**TOTAL**	**11/ 67**

Supplementary Table 5: Organ systems used to summarize symptoms.

**1. Neurology and development**
**2. Skin and accessory skin organs**
**3. Craniofacial symptoms**
**4. Eyes**
**5. Ears**
**6. Oral cavity and mouth**
**7. Cardiovascular system**
**8. Pulmonary system**
**9. Gastrointestinal system**
**10. Liver**
**11. Pancreas**
**12. Kidneys and urinary tract**
**13. Gonads**
**14. Skeletal system including joints and ligaments**
**15. Metabolic and endocrine system**
**16. Spleen and immunological system**
**17. Non-hematopoietic hematological complication (thrombosis, etc.)**
