# Supplementary material for: The clinical impact of copy number variants in inherited bone marrow failure syndromes
Source: NPJ Genom Med. 2017 May 10;2:18. doi: 10.1038/s41525-017-0019-2 (PMC5498150; doi:10.1038/s41525-017-0019-2)

Supplementary Figures

Copy number variants underlying inherited bone marrow failure syndromes

Nicolas Waespe, Santhosh Dhanraj, Manju Wahala, Elena Tsangaris, Tom Enbar, Bozana Zlateska, Hongbing Li, Robert J. Klaassen, Conrad V. Fernandez, Geoff D. E. Cuvelier, John K. Wu, Yves D. Pastore, Mariana Silva, Jeffrey H. Lipton, Joseé Brossard, Bruno Michon, Sharon Abish, MacGregor Steele, Roona Sinha, Mark J. Belletrutti, Vicky R. Breakey, Lawrence Jardine, Lisa Goodyear, Liat Kofler, Michaela Cada, Lillian Sung, Mary Shago, Stephen W. Scherer, and Yigal Dror.

Supplementary Figure 1: Analysis of clinical parameters including only patients with at least 5 patients in every IBMFS and at least one case with CNVs; grouped by cases with CNVs versus other mutations. A. Age at presentation, color bars representing corresponding median; **B.** Age at diagnosis, color bars representing corresponding median; **C**. Non-hematological organ system involvement, color bars representing corresponding median; **D.** Developmental delay; **E.** Short stature.

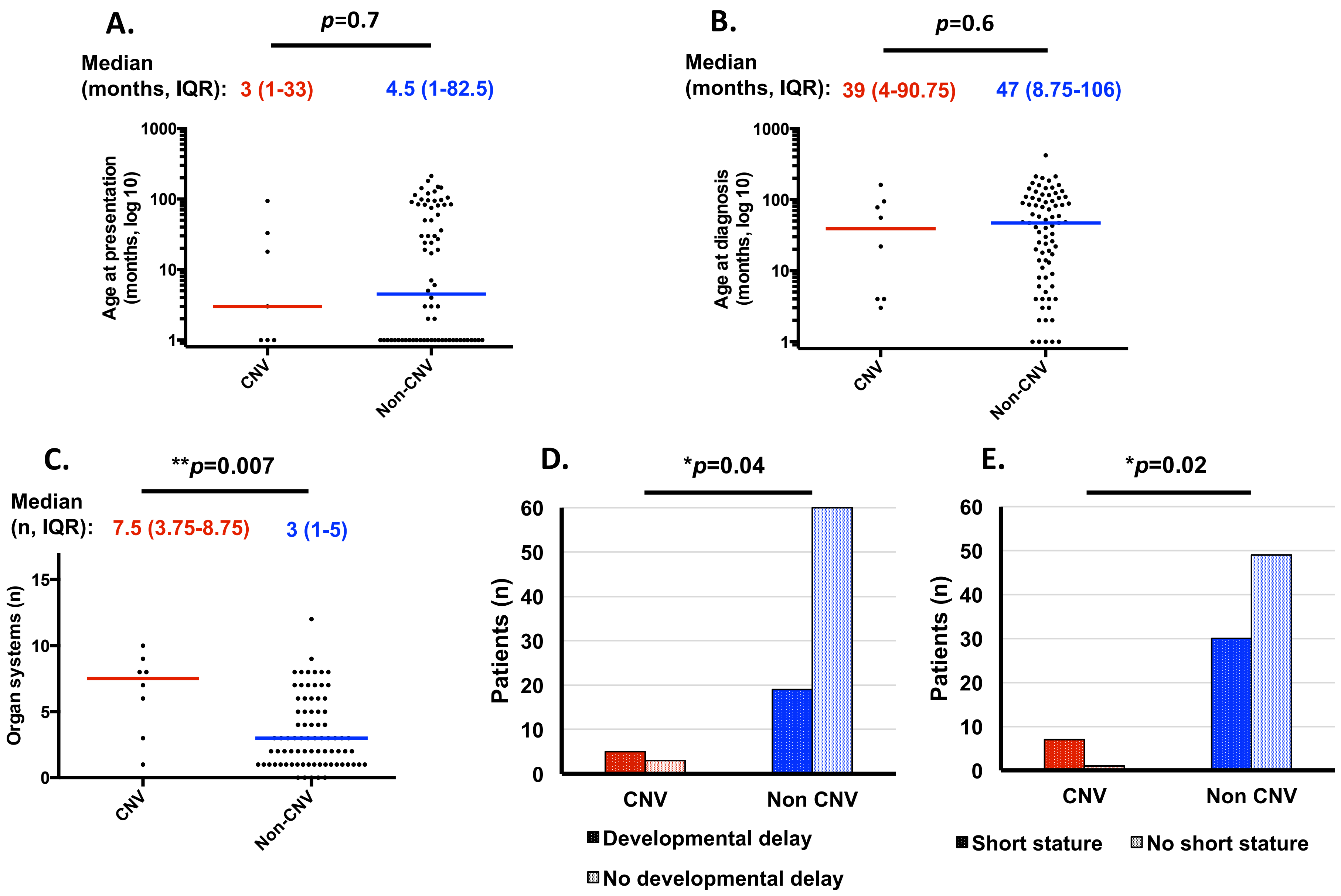

Supplementary Figure 2: Analysis of clinical parameters including only patients with at least 5 patients in every IBMFS and at least one case with CNVs; grouped by cases with CNVs versus other mutations. A. Age at onset of severe cytopenias; **B.** Overall survival.

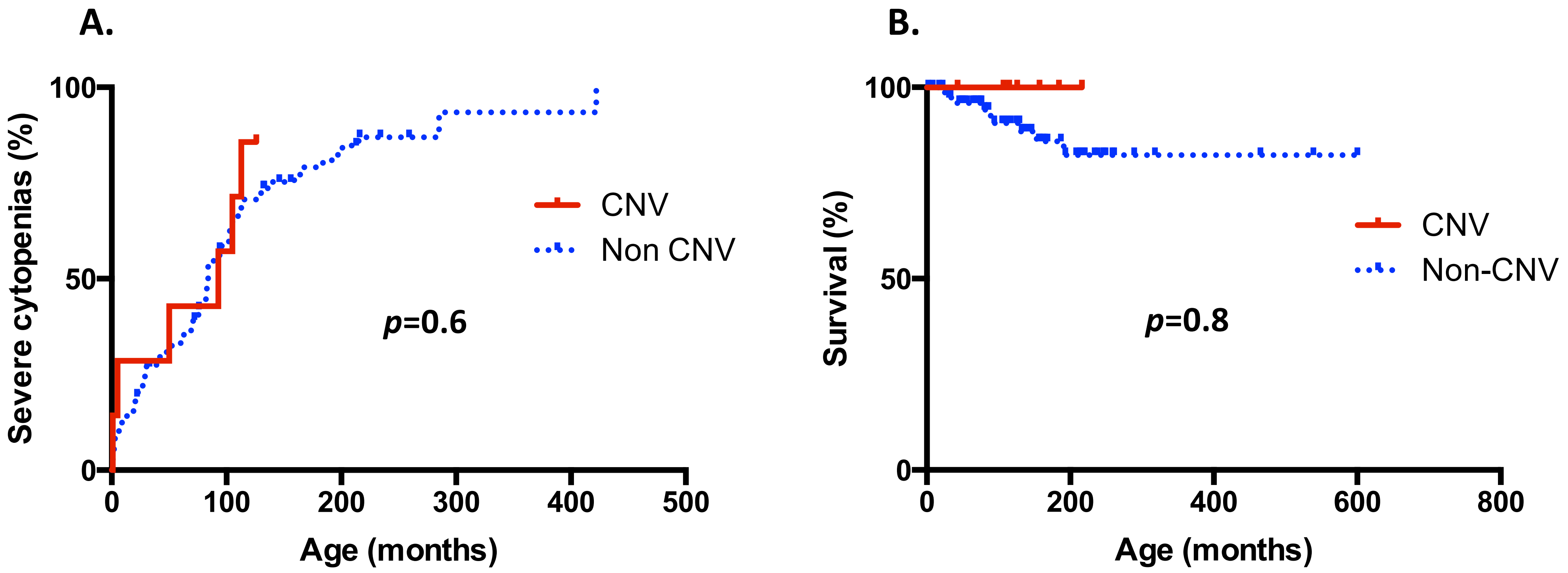

Supplement: Supplementary file 2 — Supplementary Figures [file 41525_2017_19_MOESM2_ESM.pdf]
